# Implantable Ion‐Selective Organic Electrochemical Transistors Enable Continuous, Long‐Term, and In Vivo Plant Monitoring

**DOI:** 10.1002/advs.202504283

**Published:** 2025-08-12

**Authors:** Sanggil Han, Dalila Pasquini, Mathias Sorieul, Miguel H. Boratto, Luke Gatecliff, Alan Dickson, Suhyeon Jang, Stephanie Davy, George G. Malliaras, Yi Chen

**Affiliations:** ^1^ Department of Nano‐Bioengineering Incheon National University Incheon 22012 Republic of Korea; ^2^ Center for Brain‐Machine Interface Incheon National University Incheon 22012 Republic of Korea; ^3^ Scion Group Bioeconomy Science Institute Titokorangi Drive, Private Bag 3020 Rotorua 3046 New Zealand; ^4^ Electrical Engineering Division Department of Engineering University of Cambridge 9 JJ Thomson Ave Cambridge CB3 0FA UK

**Keywords:** implantable ion sensors, in vivo plant monitoring, organic electrochemical transistors, real‐time ion recording

## Abstract

The development of plant‐specific biosensors holds the potential to uncover new insights into plant physiology and advance precision agriculture. Current sensing platforms mainly focus on broad plant phenotypes (e.g., elongation and hydration) and local environmental monitoring (e.g., temperature and moisture). Here, an ion‐selective organic electrochemical transistor (IS‐OECT) is introduced that enables real‐time monitoring of variations in potassium ion concentration within the xylem of pine trees. This work demonstrates that the high sensitivity of the IS‐OECT enables the detection of subtle variations in potassium ion concentrations in the xylem sap of living trees, and the high stability of the sensor allows for in vivo measurements over five weeks. Furthermore, the implantable sensors are fabricated using processes that are compatible with low‐cost manufacturing (i.e., lithography‐free). This sensing technology, therefore, has great potential to be a game‐changer in precision forestry and could extend to precision agriculture and horticulture practices.

## Graphical abstract

This work explores ion‐selective organic electrochemical transistors for real‐time, long‐term potassium monitoring in the xylem sap of living trees. This bioelectronic approach provides new insights into plant physiology and has the potential to revolutionize forest, agricultural, and ecological monitoring for smarter, more sustainable plant management.

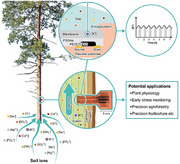

## 1 Introduction

Humanity faces the pressing challenges of climate change, biodiversity loss, and a growing global population,^[^ ^1^, ^2^ ^]^ and this necessitates a rethinking of land use. Agriculture and forestry practices need to become more sustainable and productive to secure the global supply of food and biomaterials.^[^ ^3^ ^]^ Although current precision agriculture and forestry practices leverage various monitoring technologies, they are primarily limited to assessing soil and environmental conditions (e.g., temperature and moisture) and plant phenotypes (e.g., height and leaf area).^[^ ^4^, ^5^ ^]^ These approaches offer limited capabilities for real‐time monitoring of plant health and often fail to detect the onset of stresses. To address this gap, wearable/implantable sensors have emerged as promising tools for the real‐time monitoring of ions, phytochemicals such as volatile organic compounds, and hormones.^[^ ^6^ ^]^ These sensors could enable crop growth optimization, fertilizer use efficiency, and early disease detection.

Several strategies have been developed to design plant‐specific biosensors. For example, a surface‐enhanced Raman scattering nanosensor was reported to monitor stress‐related molecules in the intercellular space of leaves.^[^ ^7^ ^]^ Plant surfaces such as leaves and stems can host wearable biosensors that monitor volatile organic compounds and sap flow rate.^[^ ^8^, ^9^ ^]^ Additionally, real‐time monitoring of sucrose in xylem sap has been achieved using implantable sucrose sensors.^[^ ^10^ ^]^ For continuous monitoring of ion transport in plants, a positron emission tomography (PET) scanner was used.^[^ ^11^ ^]^ However, PET technology is complex, as radiolabeled ions are required, and the use of bulky and expensive equipment confines it to laboratory settings. Recently, a potentiometric stainless‐steel microneedle ion sensor was inserted into the xylem of tomato plants to monitor K^+^ ion concentration, but it was only operational for a short term (one day) and failed to capture meaningful changes.^[^ ^12^ ^]^ To provide relevant data to the fields of plant physiology and precision agriculture, these ion sensors must have high sensitivity to detect subtle ion fluctuations and be capable of providing real‐time in vivo monitoring over extended periods. Only with these attributes can variations in daily ion patterns be accurately revealed. However, so far, ion sensors suitable for real‐world deployment in long‐term, continuous monitoring of ions in living plants have not yet been developed, leaving real‐time plant physiology largely unexplored.

We hypothesized that organic electrochemical transistors (OECTs) could overcome these technical limitations, due to their high signal amplification, biocompatibility and high stability.^[^ ^13^, ^14^ ^]^ OECTs use organic mixed ionic‐electronic conductors, typically poly(3,4‐ethylenedioxythiophene):polystyrene sulfonate (PEDOT:PSS), as the channel layer. In this configuration, de‐doping occurs throughout the bulk of the material via cations from the electrolyte (i.e., biofluids). Selective detection of the biofluid cations can be achieved by using an ion‐selective membrane (ISM), where an internal ion reservoir is typically placed between the ISM and the channel to enhance ionic interactions.^[^ ^15^, ^16^, ^17^ ^]^ Notably, ion‐selective OECTs (IS‐OECTs) have shown a super‐Nernstian response, providing superior sensitivity over electrode‐based sensors, along with long‐term shelf stability.^[^ ^6^ ^]^ Various OECT architectures, such as those employing extended gate^[^ ^18^ ^]^ or floating gate^[^ ^19^ ^]^ configurations, have also been developed for highly sensitive ion detection. Furthermore, selective ion detection from extracted plant sap has been successfully demonstrated using fully printed IS‐OECTs.^[^ ^17^ ^]^

Herein, we present a miniaturized IS‐OECT implanted into the xylem of living plants, enabling continuous, long‐term monitoring of specific ions. As a proof of concept, we selected the K^+^ ion as the analyte of interest, as it is the most abundant cation in plants and is involved in key physiological processes such as cellular osmosis, pH maintenance, and transmembrane transport.^[^ ^20^, ^21^, ^22^ ^]^ We selected *Pinus* spp., a widely cultivated and commercially important genus,^[^ ^23^ ^]^ as an in vivo plant model to explore the potential deployment of IS‐OECT sensing technology in nurseries for assessing plant health status. Furthermore, the predominantly xylem‐based structure of the pine trunk facilitates the implantation of sensors into the hardy trunk. We show that the sensors provide highly sensitive and stable monitoring of K^+^ dynamic variations in a living tree. This approach has the potential to reveal new insights into plant physiology and enable early stress detection, paving the way for smarter and more sustainable precision agriculture and forestry practices.

## 2 Results

### 2.1 Sensor Structure and Characteristics

**Figure 1:**
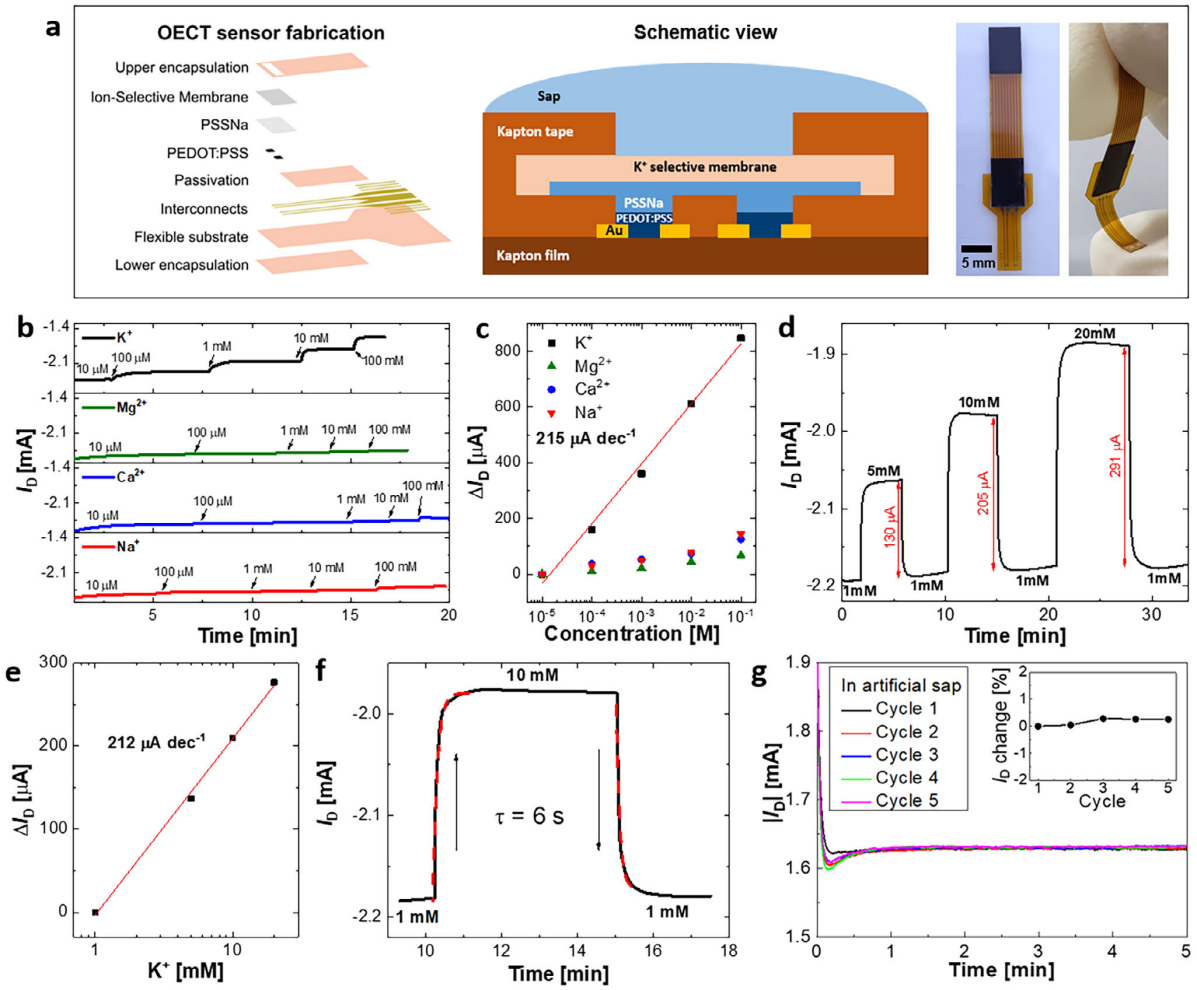
Sensor fabrication and characterization. a) Schematics of sensor fabrication, structure, and images of the fabricated sensor. b) Real‐time current response to the primary ion (K^+^) and interfering ions (Mg^2+^, Ca^2+^ and Na^+^) with different concentrations at *V*_G_ = 0 V and *V*_D_ = ‐0.4 V. c) Calibration curve of the equilibrium drain current in response to different ion concentrations. d) In vitro validation in artificial sap with repetitive changes in K^+^ concentration at *V*_G_ = 0 V and *V*_D_ = ‐0.4 V. e) Calibration curve of the equilibrium drain current obtained from (d). f) Response time of the IS‐OECT to a sudden change in K^+^ concentration from 1 to 10 mm in artificial sap. The response time is defined as the time constant obtained by fitting the data to single‐exponential functions: *I*_D_ =   − 5.89 × *e* ^*t*/τ^ − 1.98 (rising time) and *I*_D_ =  1.95 × *e* ^−*t*/τ^ − 2.17 (falling time). g) Stability test of the baseline current in artificial sap: Five cycles of 5 min current measurements with a 5 min wait time between cycles. Inset shows negligible baseline changes even after several measurements.

IS‐OECTs were fabricated on 50 µm thick flexible Kapton films as shown in **Figure** **1**a. Here, a plasticized polyvinyl chloride (PVC) membrane, containing potassium ionophores, was used as an ISM to provide K^+^ ion selectivity. A thin polyelectrolyte film of poly(sodium‐4‐styrene sulfonate) (PSSNa) was coated between the OECT channel (PEDOT:PSS) and the ISM as an internal solid electrolyte. The mobile Na^+^ ions in the PSSNa electrochemically modulate the doping level of the underlying channel layer, thereby changing the conductivity of the channel. The fabrication processes are detailed in Experimental Section (Sensor fabrication).

The sensors were characterized in chloride salt solutions. The drain current (*I*_D_) was continuously measured at constant gate and drain voltages of *V*_G_ = 0 V and *V*_D_ = ‐0.4 V, while the concentrations of the primary ion (K^+^) and interfering ions (Mg^2+^, Ca^2+^ and Na^+^) were increased in the corresponding chloride salt solutions (10^−5^ m) in a stepwise manner. As shown in Figure 1b, while an increase in the K^+^ concentration leads to significant *I*_D_ changes, the interfering ions cause minimal *I*_D_ changes. This confirms that the interfering ions hardly bind to the potassium ionophores (valinomycin), whereas the primary ions (K^+^) form ion‐ionophore complexes in the ISM, leading to an increase in the membrane potential (*E*_m_). This, in turn, causes an increase in the effective *V*_G_ despite a constant applied gate voltage (*V*_G_ = 0 V). This leads to de‐doping of the channel, which results in a change in *I*_D_. To determine the sensitivity of the IS‐OECT, the steady‐state *I*_D_ values at each concentration from Figure 1b were used to plot the *I*_D_ calibration curve (Figure 1c). A linear fit of the semilogarithmic calibration curve shows a linear response for the K^+^ ion in a wide range of 10^−5^ to 10^−1^ m with a sensitivity of 215 µA dec^−1^. The selectivity coefficients were obtained using the matched potential method, which is based on the concentration ratio of the primary and interfering ions that induces the same *E*_m_ change.^[^ ^24^ ^]^ The selectivity was calculated to be $-\log\;k_{K,\mathrm{Na}}^{\mathrm{Pot}}=3.3$, $-\log\;k_{K,\mathrm{Ca}}^{\mathrm{Pot}}=3.4$, and $-\log\;k_{K,\mathrm{Mg}}^{\mathrm{Pot}}=3.6$ for the Na^+^, Ca^2+^, and Mg^2+^ interfering ion, respectively. This means that the sensor exhibits over 1000‐fold selectivity for the K^+^ ion over the interfering ions. Given that typical concentrations of interfering ions in pine sap are in the range of a few hundred micromolar,^[^ ^25^ ^]^ negligible interference would be expected (Figure 1c).

### 2.2 Sensor Characterization in Artificial Sap

Measurements in artificial sap solution were conducted to assess the performance and stability of the IS‐OECTs. Details on the artificial sap preparation are provided in Experimental Section. The modulation of the drain current under repetitive changes of the K^+^ concentration is shown in Figure 1d. The sensor shows almost identical *I*_D_ when returning to the previous concentrations, which indicates good reversibility. The *I*_D_ calibration curve measured with the artificial sap (Figure 1e) shows a high K^+^ ion sensitivity of 212 µA dec^−1^, comparable to that measured without the interfering ions (Figure 1c). Furthermore, the response time of the sensor was determined to be 6 s (Figure 1f). Here, the response time was defined as the time constant obtained by fitting the curve to a single‐exponential function. Finally, to assess sensor stability, the *I*_D_ baseline was measured five times in an artificial sap solution containing the typical concentrations of interfering ions (Figure 1g). The IS‐OECT shows negligible *I*_D_ changes even after several cycles, which confirms that the sensors maintain a highly stable baseline in the artificial sap.

### 2.3 K^+^ Measurements In Vivo

**Figure 2:**
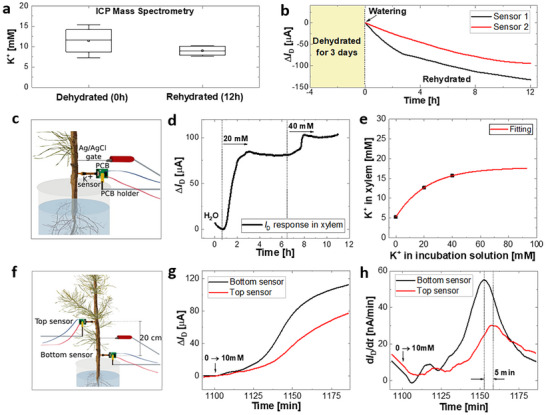
In vivo validation of sensors. a,b) Changes in K^+^ concentrations in xylem sap after 3 d of dehydration followed by rehydration: a) K^+^ concentrations before and after rehydration, obtained from sap analysis using ICP mass spectrometry. The average and standard deviation are shown in a) (*n* = 4), and b) real‐time changes in drain current measured by two different sensors implanted in two separate plants. c) Schematic of an ion sensor inserted into the xylem of a pine tree with roots directly in contact with water. d) Real‐time current response of the sensor implanted in the xylem to changes in K^+^ concentration in the incubation solution (0 → 20 → 40 mm). e) Relationship between the K^+^ concentration in the incubation solution and the xylem sap. f) Schematic of the sensors inserted 20 cm apart along the stem, with the Ag/AgCl gate positioned between them. g) Real‐time current response measured by the two sensors as the K^+^ concentration in the incubation solution was increased from 20 to 40 mm. h) Corresponding derivatives showing the time delay in K^+^ ion detection between the two sensors.

To perform in vivo validation of the sensors, we initially investigated the range of K^+^ concentration in the xylem of pine plantlets and the effect of hydration on the concentration. Eight radiata pine plantlets were kept at room temperature under natural light without watering for 3 d. After 3 d, four plantlets were cut down and the sap of their xylem collected via centrifugation. The remaining plantlets were watered, and their sap extracted 12 h after irrigation. The cation content of the extracted sap was measured by ion chromatography‐inductively coupled plasma mass spectrometry (IC‐ICP‐MS). The results obtained by IC‐ICP‐MS analysis reveal a reduction in the K^+^ concentration from 11.5 ± 3.5 (pre‐hydration) to 9 ± 1.3 mm (12 hours post‐hydration) (**Figure** **2**a). This decrease can be explained by the absorption of water leading to the dilution of K^+^ ions in the xylem sap. Following a similar protocol two sensors were implemented into the xylem of two plantlets (one per plant) just before irrigation. Figure 2b shows the *I*_D_ recorded from these two sensors over a period of 12 h after watering. The changes in *I*_D_ indicate a reduction in K^+^ ions in the xylem sap. These results confirmed the results obtained via destructive sampling and reveal that the sensors are able to detect ≈2.5 mm variation in vivo.

Additionally, a second in vivo validation test was performed using pine saplings with roots immersed in water, which allowed for a quick response to potassium supply while avoiding the buffering effect of the soil. To test this, new plants were unpotted, the soil was gently washed off their roots, and the bare root systems were submerged in a beaker filled with deionized (DI) water. Then, the ion sensor and the Ag/AgCl gate were implanted in the xylem as shown in Figure 2c. While *I*_D_ was continuously recorded, the K^+^ concentration in the incubation solution was increased to 20 and 40 mm by adding concentrated KCl solutions. The addition of K^+^ ions led to a change in *I*_D_ in a stepwise manner (Figure 2d). In this case, the K^+^ concentration in the xylem was estimated using the fitted line from the in vitro calibration curve (Figure 1e) and the *I*_D_ measured in real‐time during the experiment (Figure S1, Supporting Information). Figure 2e shows the relationship between the K^+^ concentration in the incubation solution and the estimated concentration in the xylem sap with a fitted line (Note S1, Supporting Information). The fitted line indicates the saturation of the K^+^ concentration in the xylem sap at approximately 18 mm, despite an increase in the K^+^ concentration in the incubation solution, which is likely due to the saturation of ion uptake by the sapling.

#### 2.3.1 Spatially Resolved K^+^ Measurements In Vivo

As the xylem sap flows upward, we hypothesized that the uptake of K^+^ ions would first be detected in the lower part of the stem. To test this hypothesis, two sensors were inserted 20 cm apart along the stem of one sapling, with the Ag/AgCl gate positioned between them, as shown in Figure 2f. The *I*_D_ values were simultaneously recorded by the two sensors while the K^+^ concentration in the incubation solution was increased from 20 to 40 mm. As expected, Figure 2g shows that the *I*_D_ (black curve) measured by the sensor in the lower part of the stem changed first. By plotting the derivatives of the measured *I*_D_ curves (Figure 2h), the response delay between the two sensors was estimated to be approximately 5 min. This delay is consistent with the typical sap flow rates reported for conifers (1–2 m h^−1^).^[^ ^26^, ^27^ ^]^ This confirms that our sensor setup is sensitive enough to capture these variations and can be used to study the real‐time physiological responses of plants to changes in environmental conditions or ion concentrations. Thus, these results confirm that the IS‐OECTs function properly in vivo.

#### 2.3.2 Long‐Term K^+^ Monitoring In Vivo

**Figure 3:**
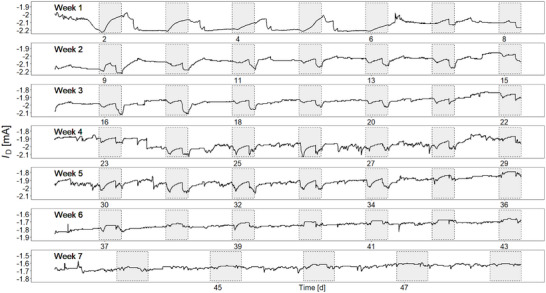
Long‐term in vivo measurement. Real‐time monitoring of K^+^ variations in a living tree over a long period (47 d). The *I*_D_ changes here represent variations in K^+^ concentration, and the gray bands indicate when the light was turned off in the growth room.

To investigate the daily changes in K^+^ concentration, a long‐term in vivo measurement was performed. A radiata pine plantlet (≈120 cm tall) was acclimatized for one month in a growth room with a controlled environment (16 h light photoperiod, 8‐hour darkness, 55–60% relative humidity at 20 °C). Once the sensor was inserted into the xylem of the plantlet (≈10 mm in diameter) along with the Ag/AgCl wire, the *I*_D_ was continuously measured at 1 min intervals, as shown in **Figure** **3**. The long‐term in vivo data reveal two distinct patterns in K^+^ variation. A notable diurnal pattern was observed in the 2^nd^ week (days 6 to 13). The K^+^ concentration increased at the onset of light, peaked around midday, and gradually decreased towards the end of the day. This diurnal pattern is strikingly similar to the K^+^ concentration changes observed in *Vicia faba* guard cells.^[^ ^28^ ^]^ Another key feature is the daily occurrence of a nocturnal peak (days 7 to 41). This peak could be related to the role of K^+^ ions in promoting leaf expansion, which primarily occurs at night when the availability of photosynthesis‐derived carbohydrates is highest.^[^ ^29^ ^]^ Perhaps, the most striking result we obtained here is that the IS‐OECT has a remarkably long lifetime (≈5 weeks) within the xylem. However, after 5 weeks of in vivo measurement, the *I*_D_ variations recorded by the sensor decreased drastically. For example, the diurnal patterns flattened, and the nocturnal peaks disappeared. To provide a more quantitative analysis of the decrease in sensitivity, the degree of *I*_D_ variations was quantified by generating weekly boxplots using the daily root mean square deviation (RMSD)^[^ ^30^ ^]^ (Figure S2, Supporting Information). The RMSD values decreased starting from the 6^th^ week, indicating that the sensor sensitivity was degraded. However, considering its cost‐effective fabrication method (i.e., entirely lithography‐free; see fabrication details in Experimental Section), the sensor lifetime of several weeks is sufficient for deployment in the fields of precision agriculture and forestry.

### 2.4 Sensor Integration and Long‐Term Wound Response

**Figure 4:**
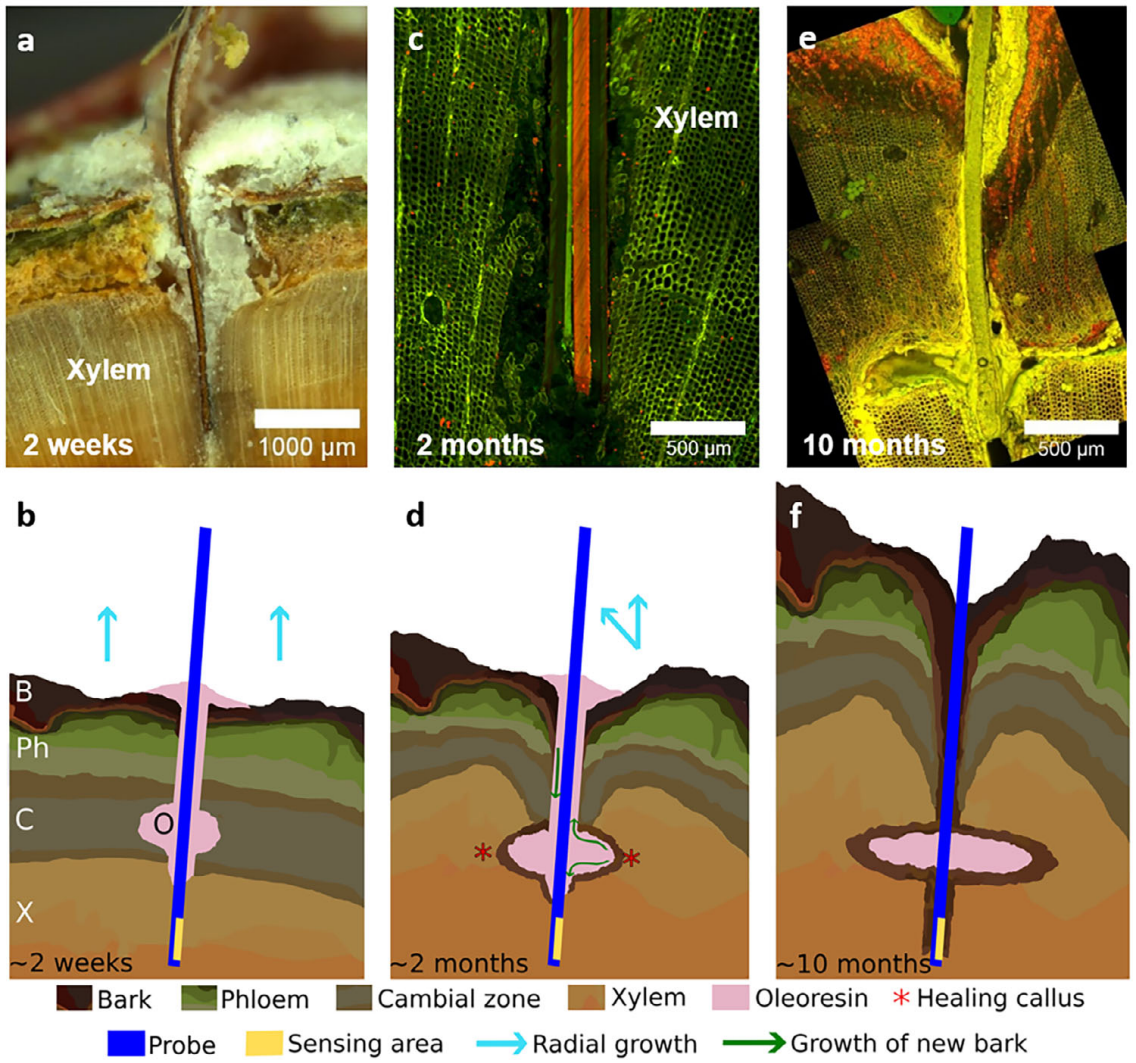
Wound response to implanted sensors. a–f) Images and schematics of wound response: Bright field image taken a) two weeks, confocal images taken c) two months and e) 10 months after sensor implantation; Schematics of the wound healing and sensor isolation processes (b,d,f)). B: Bark, Ph: Phloem, C: Cambium, X: Xylem, O: Oleoresin.

A microscopy technique was used to investigate how the sensors integrate with the pine xylem and how the living tissues respond to the wound. Blank substrates, similar in thickness to the implantable sensors, were inserted into several pine trunks. The trunks were collected two weeks, two months, and 10 months after implantation and examined using a confocal microscope. In pine species, the initial response to a deep injury is known to be resinosis.^[^ ^31^ ^]^ Indeed, after two weeks, the wound was filled with oleoresin (**Figure** **4**a,b), and the tracheids surrounding the wound are also partially filled with some slightly auto‐fluorescent material (oleoresin or tannins) (Figure S3, Supporting Information). Figure 4c illustrates that the substrate is still well integrated with the xylem two months after implantation. In addition, the cambium twists inward to initiate the formation of a healing callus, thereby creating an inner cambial zone within the wound. The remnants of this healing callus form an invagination, as seen in Figure 4d (marked with an asterisk). After several months, the bark on the exterior of the stem has grown inward and merged with the bark originating from the internal cambial zone. This newly formed bark eventually isolates the sensor from the xylem (Figure 4e,f). Microscopy analysis confirms that the sensing interface of the IS‐OECT sensor remains in direct contact with the xylem even two months after implantation (Figure 4c). This suggests that the observed decrease in sensing signal after five weeks of in vivo measurement is more likely due to an alteration of the sensing capacity (e.g., membrane saturation or sensor degradation), rather than the formation of a physical barrier (e.g., bark) insulating the sensor itself.

## 3 Discussion

This work explores the innovative use of IS‐OECTs as implantable sensor probes to monitor real‐time changes in ionic concentration in the xylem sap of living pine trees. This novel bioelectronic application overcomes significant limitations of traditional sap analysis methods, which rely on destructive sampling, labor‐intensive analyte preparation, and expensive laboratory equipment like ICP mass spectrometry. The one‐time insertion of ion sensors demonstrated here enables continuous, long‐term recording of K^+^ concentrations over several weeks, opening up new avenues for investigating plant physiology previously inaccessible due to technological constraints. However, we observed one limitation that needs to be addressed: *I*_D_ drift during the long‐term measurements both in vitro and in vivo (Figure S4, Supporting Information). This *I*_D_ drift may be explained by the de‐doping mechanism of the IS‐OECTs, where anion (i.e., PSS^–^) compensation occurs through K^+^‐ionophore complexes that penetrate through the ISM and Na^+^ ions injected into the channel from the PSSNa. Some cations may become trapped within the PEDOT:PSS channel layer. This could lead to a gradual decrease in channel conductivity (i.e., gradual de‐doping), which is believed to cause the *I*_D_ drift during the long‐term measurements. This drift issue can be resolved by implementing real‐time compensation through the subtraction of the drift baseline from the measured *I*_D_, as shown in Figure S5 (Supporting Information), which allows for accurate sensor readings.

The potential applications of the ion sensors are vast and commercially relevant. For example, nurseries could use the sensors to optimize tree growth and detect early signs of stress. Similarly, agronomic applications in potassium‐demanding crops such as kiwifruit and grapevines could benefit from real‐time monitoring to enhance yield and quality. To assess the applicability of this technology to grapevines, we implanted our sensor into the herbaceous stem of *Vitis vinifera* (Figure S6, Supporting Information). This preliminary experiment revealed that the vine exhibits tight regulation of potassium concentration in its sap. However, the concentration responds significantly to watering before reaching a stable state. To further adapt the technology for even smaller herbaceous plants like rice, the sensor's form factor would need to be miniaturized to fit thinner stems. Future developments could extend this technology to detect multiple ions (e.g., pH, Na^+^, NO_3_^−^, Ca^2+^) on a single platform. This would greatly enhance our understanding of plant physiological processes and drive innovation in precision agriculture. For instance, incorporating pH sensing could uncover links between pH, K^+^ concentration and hydraulic conductivity, while real‐time monitoring of the K^+^/Na^+^ balance could provide early warnings of salt stress in crops. Similarly, nitrate monitoring would allow for precise fertilizer application, optimizing inputs and reducing environmental impact.

Deploying this sensing technology in farm and forest environments will require further development of data loggers tailored to these conditions. These loggers must be compact, robust (e.g., waterproof), cost‐effective, and user‐friendly, featuring functionalities such as internal memory and wireless data transmission. Additionally, automated data processing methods will be essential to handle the large volumes of information generated by widespread sensor deployment. Machine learning algorithms could be employed to classify patterns and analyze relationships among ions, providing actionable insights. Integrating implantable IS‐OECT sensors, robust data loggers, and advanced data analytics into a unified technological solution will accelerate the commercialization of real‐time ion sensing platforms for living plants. This advancement holds the potential to revolutionize forest, agricultural, and ecological monitoring, paving the way for smarter, more sustainable plant management practices.

## 4 Experimental Section

### Materials

4‐Dodecylbenzenesulfonic acid (DBSA), (3‐glycidyloxypropyl)trimethoxysilane (GOPS), ethylene glycol, PSSNa (molecular weight ≈1000 000), PVC (high molecular weight), bis(2‐ethylhexyl)sebacate, potassium tetrakis(4‐chlorophenyl)borate (KT4ClPB), potassium ionophore I, tetrahydrofuran (THF), sodium chloride (NaCl), potassium chloride (KCl), calcium chloride (CaCl_2_), and magnesium chloride (MgCl_2_) were purchased from Sigma‐Aldrich and were used without further purification. PEDOT:PSS (Clevios PH1000) was acquired from Heraeus.

### Preparation of Polymer Blends

The PEDOT:PSS blend was prepared by adding 5 vol% ethylene glycol, 0.25 vol% DBSA and 1 vol% GOPS to a stock solution of Clevios PH1000. The PSSNa mixture was prepared by adding 1 m HCl (1 vol%), DBSA (0.25 vol%) and GOPS (1 vol%) to a 1.2 w/v% PSSNa solution. The ISM solution was prepared by mixing high molecular weight PVC (32.8 wt%), plasticizer (bis(2‐ethylhexyl)sebacate, 64.7 wt%), cation exchanger (KT4ClPB, 0.5 wt%) and potassium ionophore I (2 wt%) in THF.

### Sensor Fabrication

The ion sensors were fabricated in a fully lithography‐free manner. Specifically, a 50 µm Kapton film was used as a flexible substrate, which was mounted on a glass slide for easy handling during the entire fabrication process. A shadow mask for metalization was made by laser‐cutting a Kapton film (VLS3.60DT, Universal Laser Systems), and taped to the substrate. After surface activation by oxygen plasma (Diener Electronic Femto), a Ti (5 nm)/Au (100 nm) layer was deposited using an e‐beam evaporator (PVD‐75, Kurt J Lesker) and patterned by the shadow mask to form the electrodes and interconnects. The interconnects were passivated with a Kapton tape, leaving open areas to define the OECT channels. After another surface activation by oxygen plasma, the PEDOT:PSS blend, filtered through a 0.45 µm polytetrafluoroethylene (PTFE) filter, was spin‐coated on the sample at 3000 rpm. The sample was baked at 110 °C for 60 min on a hotplate to crosslink the PEDOT:PSS film, followed by immersion in DI water overnight to remove any excess compounds. The PSSNa mixture was spin‐coated on the PEDOT:PSS channels at 1000 rpm to form the internal ion reservoir. The PSSNa film, containing GOPS, was crosslinked by baking at 110 °C for 60 min and subsequently immersed in a 100 mm NaCl solution overnight to retain Na^+^ ions in the film while removing excess compounds. The ISM solution was drop‐cast onto the PSSNa film and dried at room temperature to form the ISM film. Note that the ISM layer must completely cover the PSSNa layer; otherwise, interfering ions can pass through the exposed PSSNa, potentially leading to false analyte readings. Finally, the sensors were encapsulated with Kapton tape, leaving the sensing area open to ensure that only the sensing part is exposed to the pine xylem sap.

### Sensor Characterization

The contact pads on the fabricated sensors were bonded to a flexible flat cable (FFC) using a bonder (FINEPLACER pico2, Finetech GmbH) and an anisotropic conductive film (ACF, 5 µm particulate, 3TFrontiers). The FFC was then connected to a printed circuit board (PCB) using a zero insertion force (ZIF) connector. All electrical measurements were performed using either a semiconductor parameter analyzer (Keithley 4200A‐SCS) or a precision source/measure unit (SMU, Keysight B2902B). To obtain real‐time I_D_ responses to the primary ion (K^+^) and interfering ions (Na^+^, Ca^2+^ and Mg^2+^), the sensor was immersed in the corresponding chloride salt solutions (10^−5^ m), and *I*_D_ was recorded with an Ag/AgCl (3 m NaCl) as a gate electrode at *V*_G_ = 0 V and *V*_D_ = ‐0.4 V. After the initial current stabilized, ion concentrations were increased by successive additions of the corresponding salt solutions (KCl, NaCl, CaCl_2_, and MgCl_2_) in the range of 10^−5^ to 10^−1^ m. For in vitro validation, artificial sap was prepared by dissolving typical concentrations of interfering ions (0.2 mm Na^+^, 0.9 mm Ca^2+^, and 0.3 mm Mg^2+^) found in pine xylem sap in DI water.^[^ ^25^ ^]^ The sensor was immersed in the artificial sap with an Ag/AgCl wire, and I_D_ was continuously recorded at *V*_G_ = 0 V and *V*_D_ = ‐0.4 V while repetitively changing the K^+^ concentration.

### Plant Preparation

Two different pine species were selected for this study: *Pinus sylvestris* L. (Scots pine) and *Pinus radiata* D. Don (Monterey pine). *P. sylvestris* plantlets were purchased from the market and used for in vivo sensor validation with roots in water at the University of Cambridge (UK). *P. radiata* plantlets were grown from seeds. The plantlets were further grown in 1 L Ellepot paper pots containing a standard growth mixture for 18 months in Scion's nursery (Rotorua, New Zealand) and were fertilized with Peters Professional (N.P.K. 20‐20‐20) at a concentration of 1 g L^−1^. The plantlets were then used for in vivo experiments with roots in soil, and to collect sap analysis and microscopy data at Scion.

### Sensor Validation In Vivo

An incision was made on the stem using a scalpel, and a mini spatula was used to enlarge the incision. Artificial sap (≈200 µL) was pipetted into the incision to minimize embolism, after which the ion sensor was inserted into the xylem. Likewise, an Ag/AgCl wire (diameter ≈0.5 mm) was inserted as a gate electrode into the trunk through a hole created with a needle. The sensor and Ag/AgCl wire were connected to the SMU, and the *I*_D_ was continuously recorded at *V*_G_ = 0 V and *V*_D_ = ‐0.4 V. For in vivo sensor validation with roots submerged in water, *P. sylvestris* plantlets were unpotted, and their bare roots were placed in DI water. After *I*_D_ stabilized, concentrated KCl solutions were added successively to increase the K^+^ concentration to 20 and 40 mm. *P. radiata* plantlets were used for in vivo tests with roots in soil. First, after 3 d of dehydration followed by rehydration, the ion sensors were inserted into the xylem, and *I*_D_ was recorded for 12 h to monitor changes in the K^+^ concentration. The long‐term in vivo measurement was performed in a growth room with a controlled environment. The room temperature was set to 20 °C, with a light period from 6 am to 10 pm (16 h) and a dark period of 8 hours. A light‐emitting diode (LED) (HL26‐P02‐03) was used as the light source which provides extended photosynthetically active radiation (ePAR) with photon flux of ≈120 µmol s^−1^. The variation in K^+^ concentration was monitored using the implanted sensor over a period of 7 weeks.

### Sap Preparation and Analysis

Plant stems were cut and debarked, and the cambium was scraped away with a blade. The bare xylem stems were cut into 4 cm pieces and placed in a 50 mL centrifuge tube. The tubes were centrifuged at 10 000 rpm for 15 min. The extracted sap was analyzed by IC‐ICP‐MS.

### Microscopy

The wound response to probe insertion was evaluated on two‐year‐old, closely spaced *P. radiata* trees, approximately 5 cm in diameter, growing at Scion. A vertical cut was made using a razor blade, after which dummy probes were immediately inserted and left in place until harvest. The entire cross‐section of the stem was processed during harvesting. Samples were either examined fresh (stained with 0.0002% acriflavine) or after resin embedding^[^ ^32^ ^]^ using a confocal microscope (Leica SP5). The excitation wavelengths used were 355 and 488 nm, with emission bandwidths of 380–480, 493–538, and 625–800 nm.

## Conflict of Interest

The authors declare no conflict of interest.

## Author Contributions

S.H. and D.P. contributed equally to this work. Y.C. and G.G.M. conceived the idea. G.G.M. supervised the project. S.H. designed and fabricated the implantable ion sensors with the help of L.G. and M.H.B. S.H. and L.G. performed the characterization of the sensors. S.J. and M.H.B. carried out in vitro validation (Figure 1) and S.H., M.S., and Y.C. performed in vivo sensor validation (Figure 2). D.P., M.S. and Y.C. performed the in vivo long‐term test (Figure 3). M.S. prepared the samples for ICP mass spectrometry. A.D and S.D. conducted the investigation of long‐term wound response (Figure 4). S.H., D.P., M.S., and Y.C. wrote the manuscript, and all authors contributed to the review and revision of the manuscript.

## Supporting information



Supporting Information

## Data Availability

The data that support the findings of this study are available from the corresponding author upon reasonable request.
