# Supplementary material for: Implantable Ion‐Selective Organic Electrochemical Transistors Enable Continuous, Long‐Term, and In Vivo Plant Monitoring
Source: Adv Sci (Weinh). 2025 Aug 12;12(41):e04283. doi: 10.1002/advs.202504283 (PMC12591102; doi:10.1002/advs.202504283)
Supplement: Supplementary file 1 — Supporting Information [file ADVS-12-e04283-s001.docx]

Supporting Information

**Implantable Ion-Selective Organic Electrochemical Transistors Enable Continuous, Long-Term and in vivo Plant Monitoring**

Sanggil Han*, Dalila Pasquini, Mathias Sorieul, Miguel H. Boratto, Luke Gatecliff, Alan Dickson, Suhyon Jang, Stephanie Davy, George G. Malliaras*, and Yi Chen*

**Contents**

| **Note 1** | Method used to determine the relationship between the K^+^ concentration in the incubation solution and the xylem sap. |
| --- | --- |
| **Figure S1** | K^+^ concentrations in the xylem, as estimated from the measured *I*_D_. |
| **Figure S2** | Degree of *I*_D_ variations during the long-term in vivo measurement. |
| **Figure S3** | Microscopic analysis of auto-fluorescent material generated around the wound. |
| **Figure S4** | *I*_D_ drift observed in long-term measurements. |
| **Figure S5**  **Figure S6** | *I*_D_ drift compensation.  Preliminary in vivo measurement in *Vitis vinifera*. |

**Note 1:** Method used to determine the relationship between the K^+^ concentration in the incubation solution and the xylem sap.

First, the measured *I*_D_ was converted into the K^+^ concentration as shown in Supplementary Figure 1. Specifically, the initial K^+^ concentration in xylem sap at the start of the measurement was assumed to be the average K^+^ concentration (5.8 ± 1.36 mM) of sap samples extracted from 8 two-year-old pines, as measured by ICP-MS. Then, the K^+^ concentrations were estimated using the fitted line from the in vitro calibration curve (Fig. 1e) and the measured *I*_D_ (Fig. 2d). The K^+^ concentrations indicated by the red dashed lines in Supplementary Figure 1 were plotted in relation to the K^+^ concentration in the incubation solution (See Fig. 2e). The fitted line in Fig. 2e was generated using the function,$f\left( x \right)=5.27+12.5\times(1-{0.956}^{x})$.

**Figure S1.** K^+^ concentrations in the xylem, as estimated from the measured *I*_D_. Changes in the K^+^ concentration in the xylem sap in response to a sudden increase (0 → 20 → 40 mM) in K^+^ concentration in the incubation solution, in which the roots were submerged.


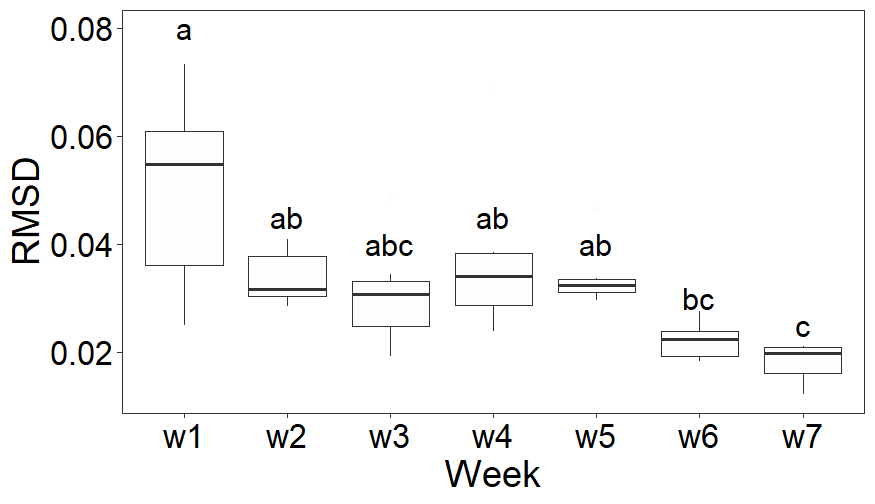


**Figure S2.** Degree of *I*_D_ variations during the long-term in vivo measurement. The boxplots were generated using the root mean square deviation (RMSD), *n* = 7. Here, RMSD was calculated as the square root of the mean of the squared differences between the vertical deviations of the actual measurements (i.e., measured *I*_D_ values) and the expected measurements (i.e., the daily mean line) (*dplyr* package, version 1.1.3). Boxplots with the same letters indicate that the groups are not significantly different from each other, as determined by a one-way non-parametric analysis of variance (Kruskal-Wallis test – ‘kruskal.test()’ function) followed by a post-hoc Dunn's test for pairwise comparisons (‘dunn.test()’ function from the *dunn.test* package, version 1.3.6). All statistical analyses were conducted using R software (version 4.3.1), and results were considered significant at p < 0.05.


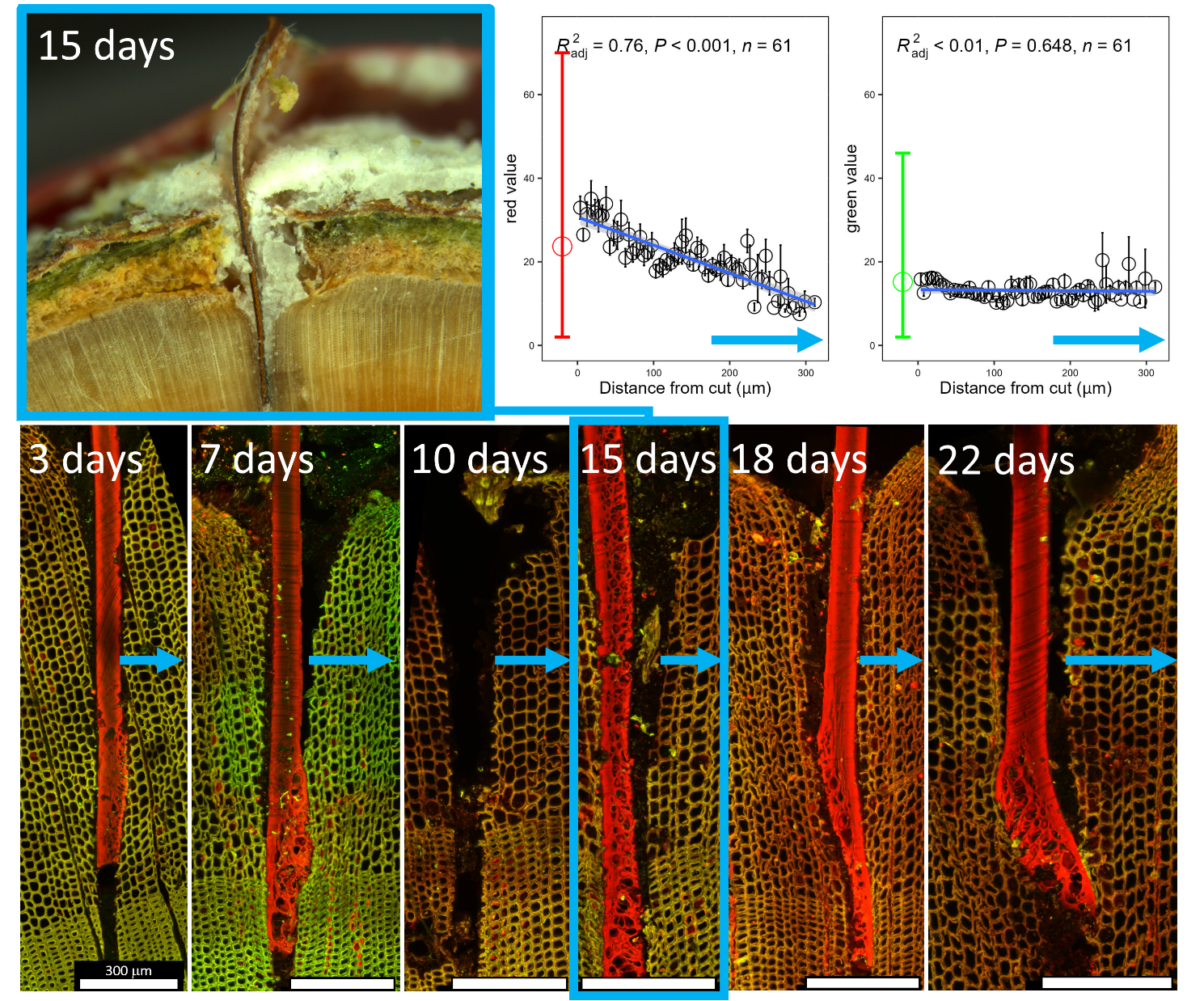


**Figure S3.** Microscopic analysis of auto-fluorescent material (oleoresin or tannins) generated around the wound. In the graphs, the error bars represent the standard error (SE) of the data, and the *x*-axis represents the distance from the cut, as indicated by the cyan arrows in the images below. The red fluorescent material in the lumen decreases with distance from the cut, while the green one remains constant with distance.


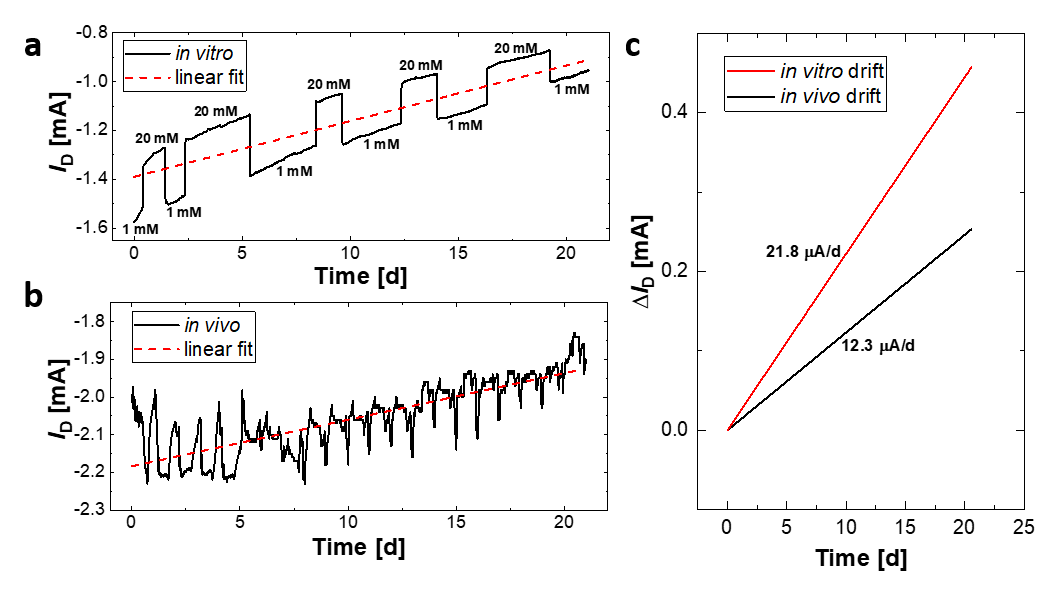


**Figure S4.** *I*_D_ drift observed in long-term measurements (3 weeks). a) In vitro measurement in artificial sap with repetitive changes in K^+^ concentration and b) in vivo measurement in the xylem of a living tree. Both were recorded at *V*_G_ = 0 V and *V*_D_ = –0.4 V. **c** *I*_D_ drift slopes extracted from the linear regression fits.

**Figure S5.** *I*_D_ drift compensation. Subtracting the drift baseline from the measured *I*_D_ can help overcome the drift issue during long-term measurements.


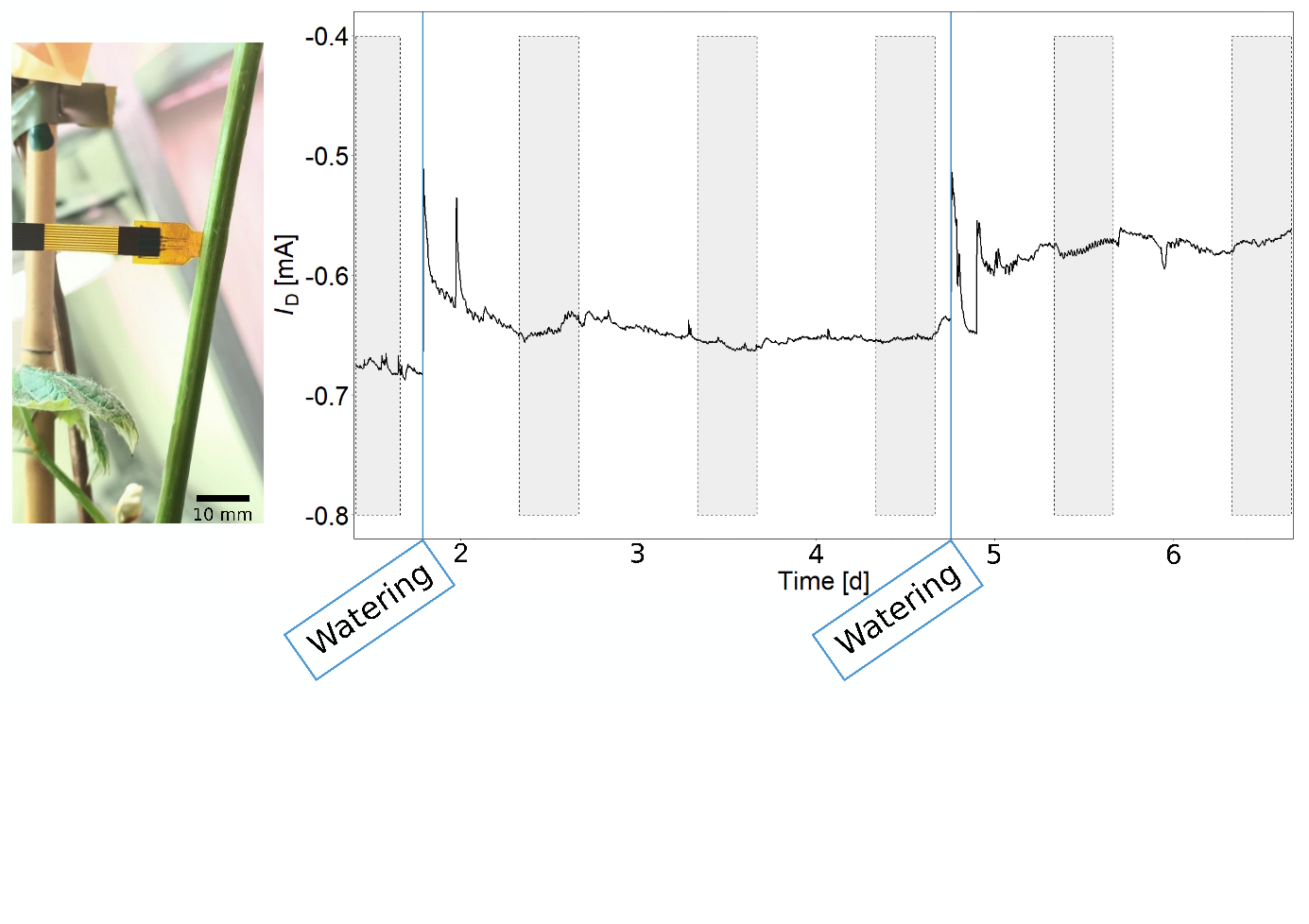


**Figure S6.** Preliminary in vivo measurement in *Vitis vinifera*. Real-time monitoring of K^+^ variations in a living vine over 6 days period. The *I*_D_ changes here represent variations in K^+^ concentration, and the gray bands indicate when the light was turned off in the growth room.
